# Association of Eyelid Margin Thickness and Meibography in Dogs With Meibomian Gland Dysfunction

**DOI:** 10.1111/vop.13326

**Published:** 2025-02-18

**Authors:** Giyeon Kim, Seonmi Kang, Junehee Seo, Kangmoon Seo

**Affiliations:** ^1^ Department of Veterinary Clinical Sciences, College of Veterinary Medicine and Research Institute for Veterinary Science Seoul National University Seoul Korea

**Keywords:** dry eye disease, eyelid margin thickness, meibography, meibomian gland dysfunction, meibomian gland loss

## Abstract

**Objective:**

To measure the eyelid margin thickness (LMT) in dogs with meibomian gland dysfunction (MGD) and evaluate its correlation with meibomian gland (MG) morphology.

**Animals Studied:**

Fifty‐nine client‐owned dogs.

**Procedure:**

The LMT was measured on slit lamp biomicroscopy images and divided into groups of 1 to 4, from the thinnest to thickest, based on quartiles. MG morphology, including distortion, thickening, shortening, and dropout, was evaluated using noninvasive infrared meibography. The LMT and meibography results were compared between the MGD and normal groups. Statistical analysis was performed to determine the correlation between LMT and MG morphology.

**Results:**

The mean LMT was significantly greater in the MGD group (1.18 ± 0.19 mm) than the normal group (1.00 ± 0.13 mm) and was positively correlated with MG loss (*p* < 0.01). The LMT was thicker in dogs over 12 years (1.25 ± 0.20 mm). The LMT group 4 (≥ 1.26 mm) had the highest percentage of abnormal MG (95.7%) and MG loss area (37%). The total abnormal MG ratio and thickened MG ratio were significantly higher in the LMT group 4. LMT ≥ 1.20 mm was identified as a potential indicator for MG loss area of more than one‐third.

**Conclusions:**

Eyelids with thick LMT had more abnormal MG morphology, including thickening and dropout. An LMT ≥ 1.20 mm could be a criterion to suspect MGD. Thus, the LMT could be a simple screening tool to predict MG loss and might aid in the diagnosis and early management of MGD with a sensitivity of 0.645 and a specificity of 0.768.

## 1 Introduction

Meibomian gland dysfunction (MGD) is one of the major causes of ocular surface disease in humans and dogs [1, 2, 3, 4, 5]. Obstructive MGD, which is the main form of primary MGD, is caused by terminal obstruction of the meibomian gland (MG) duct, leading to disuse atrophy of the gland and changes in the quantity and quality of meibum secretion [6, 7]. This causes tear film instability and evaporative dry eye disease, which presents symptoms such as irritation, discomfort, chronic inflammation and damage to the ocular surface [4, 6, 8]. As atrophic changes in the MG are influenced by the degree and duration of obstruction, early diagnosis and management is certainly beneficial, as it can delay disease progression and is more effective when initiated early [7, 9, 10].

Eyelid margin abnormality assessment, MG expression, interferometry, meibometry and meibography are diagnostic tools for MGD [11]. In the early preclinical stage, symptoms such as eyelid abnormalities may not be obvious, and MGD can only be diagnosed using meibography or gland expression for meibometry [6, 12, 13]. However, meibometry is not repeatable in veterinary clinics [14, 15], requires specialized equipment, and is somewhat invasive [11, 16].

Non‐contact infrared meibography is noninvasive and directly identifies abnormalities of MG morphologies such as distortion, thickening, thinning, shortening, and dropout in vivo. These abnormal morphologies of the MG are thought to reflect the progression of MGD [17, 18]. Therefore, meibography has been widely applied for the diagnosis of MGD in humans [3, 19, 20]. In veterinary medicine, it is relatively novel and its application is limited due to the high cost of the necessary equipment and the technical expertise required [3, 16].

In human studies, lid margin abnormalities, including thickening, have been analyzed for their association with MG loss [13, 21]. Zhou et al. found that lid margin thickening score was strongly correlated with MG loss [9]. Some human studies have directly measured eyelid thickness to establish simple and objective screening tool for MGD [22]. These studies showed that eyelid thickening, which was easily detectable with the naked eye or slit lamp biomicroscopy, could be correlated with MG loss and could help identify the need for additional and detailed examinations, such as meibography or interferometry, to diagnose MGD at an earlier stage.

Eyelid margin thickening has also been recognized as a diagnostic criterion for MGD in dogs; however, no previous studies have utilized quantitative thickness measurements to evaluate the disease [3, 16]. Thus, this study aimed to measure eyelid margin thickness in normal and MGD dogs, and analyze the correlation between lid margin thickness and abnormal MG morphology.

## 2 Materials and Methods

### 2.1 Animals

Seventy‐eight upper eyelids of 46 dogs diagnosed with MGD based on the presence of lid margin abnormalities, using criteria adapted from human standards and modified from a previous study, were included in this study [13, 16, 21]. MGD was diagnosed if one or more of the following criteria were met: (1) thickened eyelid margins, (2) irregular eyelid margins, (3) MG orifice plugging, or (4) retro‐placed MG orifices. Additionally, the study included a voluntary normal group of 22 healthy eyelids of 17 dogs without MGD. In the case of four dogs, one eye was classified as normal, whereas the other was categorized as MGD. Therefore, these dogs were included in both groups for statistical analysis.

The eyes that met the following criteria were excluded: (1) ocular/intraocular surgery within the past 3 months; (2) the presence of active inflammatory eyelid diseases (e.g., blepharitis, periocular dermatitis, chalazion, and hordeolum) that could affect lid abnormalities, or these conditions not being in remission within the past 3 months; (3) presence of irregular eyelid structures such as cicatrices, entropion, ectropion, or eyelid mass, or history of eyelid surgery to resolve these condition (e.g., mass resection, correction of entropion or ectropion, and filler injection); (4) severe conjunctival pigmentation or severe uncooperativeness/aggressiveness of the patient that limit the performance of slit lamp biomicroscopy and meibography.

### 2.2 Clinical Ophthalmic Examination

The animals were examined under gentle restraint and without sedation. All data were measured and analyzed by the same researcher (G. Kim) to prevent inter‐observer differences.

#### 2.2.1 Measurement of Eyelid Margin Thickness

**FIGURE 1:**
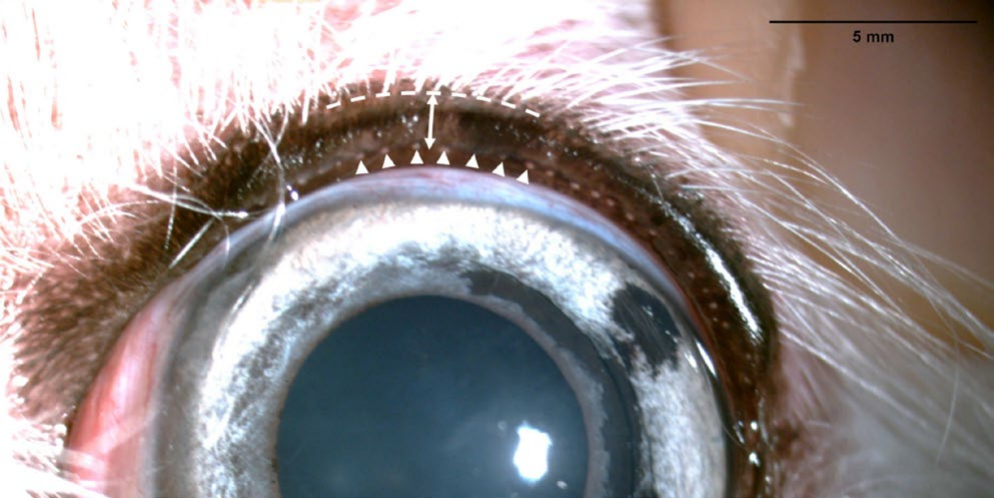
Measurement of eyelid margin thickness (LMT). LMT (↔) was measured as the length from the cilia line (– – –) to the gray line (▲) at the center of the upper eyelid.

Lid margin thickness (LMT) was defined as the distance from the cilia line to the gray line (MG orifices) in the center of the upper eyelid (Figure 1) by adopting the previous human measurement method, which measured the distance from the posterior lash line as the anterior lid margin to the mucocutaneous junction as the posterior lid margin [13, 18].

Lid margin images were obtained with slit lamp biomicroscopy (Topcon‐Model SLD7; Topcon Corp., Tokyo, Japan), under diffuse illumination at 10× magnification. The eye was positioned in the middle and the focus was adjusted on the upper eyelid margin. The eyelid margin was slightly everted with the fingers just until the gray line was visible, so the gray line and cilia line were shown in the same plane at once. Care was taken to ensure that the eversion was neither excessive nor insufficient to ensure the accuracy of the LMT measurements.

LMT was measured using the ImageJ (Bethesda, Maryland, USA) program that calibrated image pixel to millimeters from one reference image that captured the lid margin and caliper at the same focal length. This calibration was subsequently applied to measure the widths of the eyelid margins in other images. The LMT values measured in this study were divided into four groups based on quartiles: 1 = LMT equal to or less than 1.00 mm; 2 = LMT from 1.01 to 1.10 mm; 3 = LMT from 1.11 to 1.25 mm; and 4 = LMT more than 1.25 mm. The average of the two measurements was calculated and used for statistical analysis. The relationships between LMT and age and between LMT and sex were analyzed, with age categorized into four groups: 1 = equal to or less than 4 years, 2 = from 5 to 8 years, 3 = from 9 to 12 years, and 4 = over 12 years.

#### 2.2.2 Meibography

Non‐contact infrared meibography was performed using the portable OSA‐VET (OSA‐VET, SBM Sistemi, Torino, Italy). The upper eyelid was fully everted manually to assess the middle two‐thirds of the palpebral conjunctiva. The lower eyelid was excluded because of the difficulty of eversion, which often requires excessive force and causes discomfort in dogs [12]. Additionally, meibography of the lower eyelid is often omitted in humans because of its poorer inter‐examiner reliability [23]. MG loss was evaluated using the MG loss rate (MGLR), calculated using the built‐in software of OSA‐VET by comparing the MG loss area to the selected tarsal area. Among the various criteria for assessing MG loss [13, 24, 25], criteria by Arita et al. were used, and MG loss area was scored as a meiboscore from 0 to 3; 0 = no MG loss area; 1 = loss area less than one‐third; 2 = loss area more than one‐third but less than two‐thirds; and 3 = loss area more than two‐thirds [21].

**FIGURE 2:**
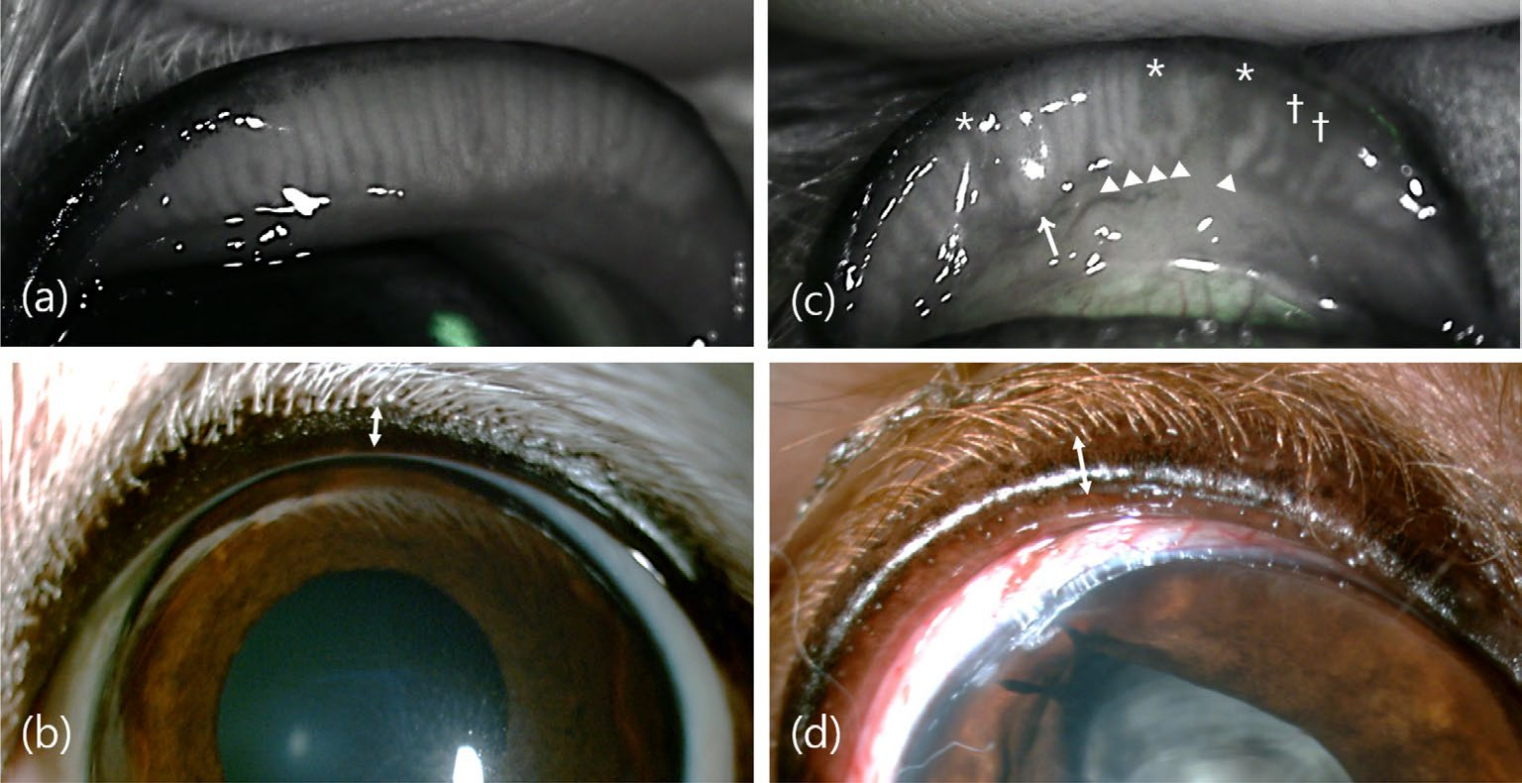
Meibography and slit lamp biomicroscopy image of normal (a, b) and meibomian gland dysfunction (c, d) dog. (a) Normal meibography with parallel MGs. (b) Thin eyelid margin (LMT) of a normal dog (↔). (c) Meibography showing MG dropout (*), shortening (†), distortion (▲), and thickening (↑). (d) Thick LMT of a dog with meibomian gland dysfunction (↔). Note the round and thickened lid margin.

Abnormal MGs, including thickened, shortened, and distorted MGs, were evaluated and counted. A thickened MG was defined as having a thickness equal to or greater than twice that of a normal MG [18]. A shortened MG was defined as having a length shorter than a normal MG. A distorted MG was defined as an angle of more than 45°, not following the parallel course of the normal glands [26] (Figure 2). The number of abnormal MGs was divided by the total number of MGs imaged in the meibography to calculate the abnormal MG ratios: thickened MG ratio, shortened MG ratio, distorted MG ratio, and total abnormal MG ratio [26]. Six eyes with 100% MG loss were excluded from calculation of abnormal MG ratio.

### 2.3 Statistical Analyses

The normality was evaluated using the Shapiro–Wilk test. The Mann–Whitney test and Fisher's exact test were performed to determine whether there were differences in age, sex, and breed between the normal and MGD groups. The correlation between the LMT and meiboscore was evaluated using Spearman's rank correlation. Student's *t*‐test was performed to compare the difference in LMT between the normal and MGD groups and between different sex groups.

Differences in LMT between age groups were analyzed using one‐way analysis of variance (ANOVA). Differences in the MGLR and abnormal MG ratio were analyzed between the normal and MGD groups and among the four LMT groups using the Kruskal–Wallis test.

The receiver operating characteristic (ROC) curve and area under the curve (AUC) were analyzed to determine the LMT threshold that would indicate MGD with optimal test sensitivity and specificity [27].

Statistical analysis was conducted using IBM SPSS Statistics version 29.0 (IBM Corp., Armonk, NY, USA), and a *p* value < 0.05 was considered statistically significant.

## 3 Results

### 3.1 Characteristics of Participating Animals

A total of 100 eyes of 59 dogs were included in the study. Of these, 78 eyes of 46 dogs were diagnosed with MGD based on lid margin abnormalities and 22 eyes of 17 dogs were included in the normal group. Four dogs had either the right or left eye included separately in the normal and MGD groups, respectively.

No statistically significant differences were observed between the two groups with respect to age (*p* = 0.15), sex (*p* = 0.27) and breed (*p* = 0.29) (Table 1). The median age of the normal and MGD groups was 9 years (range, 4–14) and 10 years (range, 1–16), respectively. A total of 15 breeds were included in the study, predominantly small breeds; the most prevalent breed was Poodles (*n* = 15), followed by Maltese (*n* = 11) (Table 2).

**TABLE 1:** Characteristics of the dogs included in the study.

	Normal	MGD	*p*
Age (years), median (range)	9 (4–14)	10 (1–16)	0.15
Sex *n* (%)			
Intact males	0 (0)	1 (2.2)	0.27
Castrated males	6 (35.3)	22 (47.8)	
Intact females	4 (23.5)	3 (6.5)	
Spayed females	7 (41.2)	20 (43.5)	
Total (*n*)	17	46	

*Note:* Median (range) of age and number (percentage) of dogs in each sex groups in normal and MGD dogs.

Abbreviation: MGD, meibomian gland dysfunction.

**TABLE 2:** Breeds of dogs included in the study.

	Normal	MGD
Poodles	5	11
Maltese	3	9
Bichon Frise	0	7
Shih‐tzu	3	4
Pomeranian	2	4
Mixed	0	4
Cocker Spaniel	0	2
Boston Terrier	1	0
Dachshund	0	1
French Bulldog	0	1
Samoyed	1	1
Shetland Sheepdog	0	1
Spitz	1	0
West Highland White Terrier	1	0
Yorkshire Terrier	0	1
Total	17	46

Abbreviation: MGD, meibomian gland dysfunction.

### 3.2 Correlation of LMT and Meibography

**FIGURE 3:**
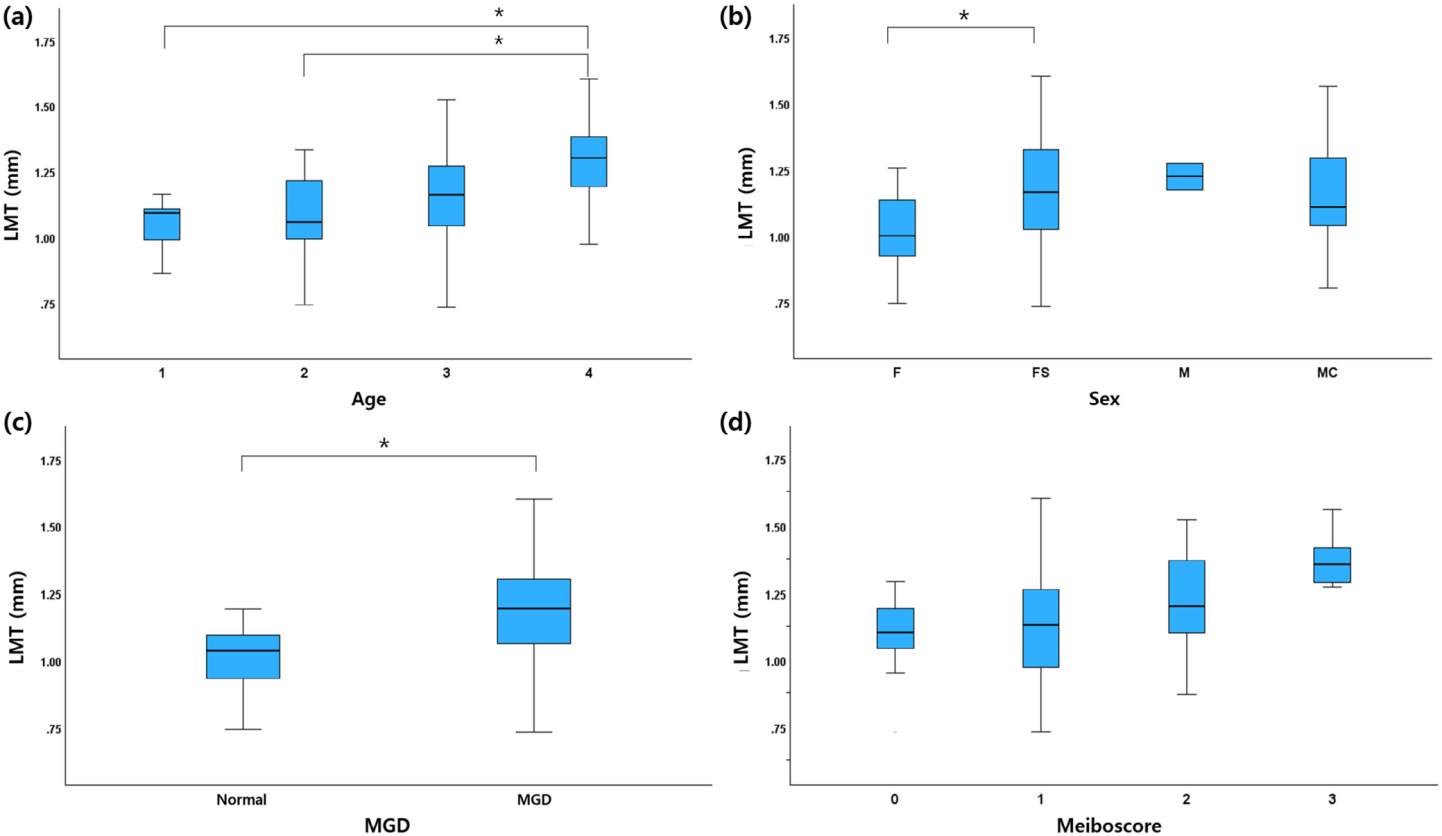
Boxplots comparing LMT of dogs in the present study. (a) age, (b) sex, (c) presence of MGD, and (d) meiboscore. Statistically significant correlation is marked with an asterisk; *p < 0.05. F, intact female; FS, spayed female; LMT, eyelid margin thickness; M, intact male; MC, castrated male; MGD, meibomian gland dysfunction.

The LMT was found to be significantly thicker in the age group over 12 years (1.25 ± 0.20 mm), than in the age groups under 4 years (1.04 ± 0.14 mm, *p* = 0.02) and under 8 years (1.08 ± 0.18 mm, *p* = 0.01) (Figure 3a). No significant difference in LMT was found between spayed females (1.17 ± 0.21 mm) and castrated males (1.14 ± 0.17 mm) groups (*p* > 0.05), but the LMT of intact females (1.03 ± 0.18 mm) was significantly thinner than that of spayed females (*p* < 0.05) (Figure 3b).

LMT was significantly different between the normal (1.00 ± 0.13 mm, mean ± SD) and MGD (1.18 ± 0.120 mm) group (*p* < 0.05, 95% confidence interval) (Figure 3c). In the MGD group, the LMT was moderately positively correlated with the meiboscore (rho = 0.404, *p* < 0.01) (Figure 3d). MGLR (*p* < 0.001), abnormal MG ratio (*p* < 0.001), thickened MG ratio (*p* = 0.007), and shortened MG ratio (*p* = 0.001) were significantly higher in the MGD group (Table 3).

**TABLE 3:** Morphology of abnormal meibomian gland of normal and dogs with meibomian gland dysfunction in the study.

	Normal	MGD	*p*
MGLR (%)	0 (0–9)	25 (0–100)	< 0.001**
Abnormal MG ratio	0.08 (0–0.31)	0.35 (0–1.50)	< 0.001**
Thickened MG ratio	0 (0–0.23)	0.08 (0–0.50)	0.007*
Shortened MG ratio	0 (0–0.07)	0.15 (0–1.00)	0.001**
Distorted MG ratio	0.02 (0–0.20)	0.09 (0–1.50)	0.07

*Note:* Median (range) of MGLR and abnormal MG ratios in normal and MGD dogs. Statistically significant correlations are marked with asterisks: *p < 0.05; **p < 0.001.

Abbreviations: MG, meibomian gland; MGD, meibomian gland dysfunction; MGLR, meibomian gland loss rate.

**FIGURE 4:**
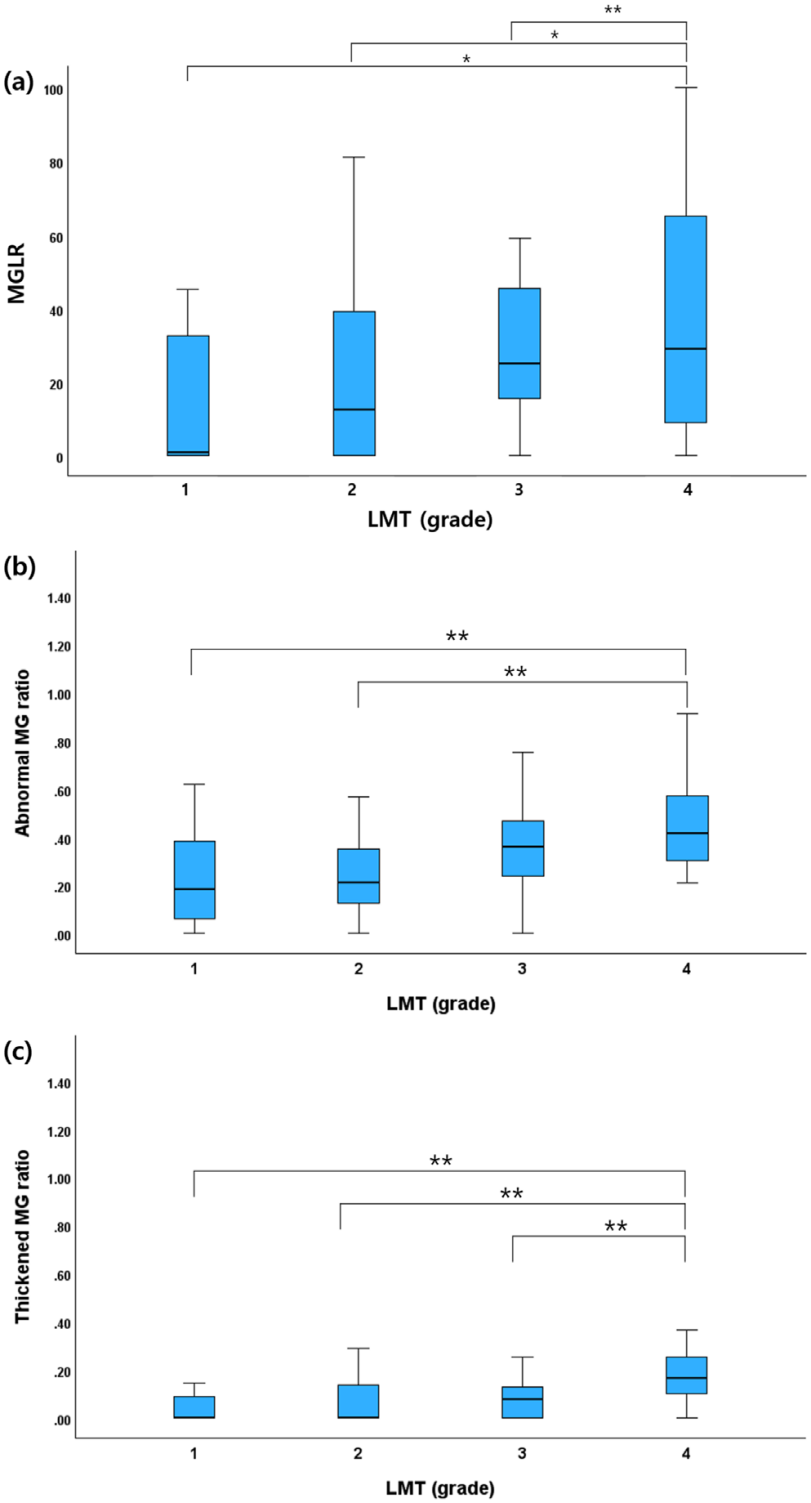
Boxplots comparing four eyelid margin thickness (LMT) grades in (a) meibomian gland loss rate (MGLR), (b) total abnormal meibomian gland ratio and (c) thickened meibomian gland ratio. Statistically significant correlation is marked by asterisk; *p < 0.001, **p < 0.01.

Comparing abnormal MG morphology between 4 groups of LMT, the percentage of eyes with abnormal MG was highest in LMT group 4 (95.7%) (Table 4). MGLR (37%, 0–100) (median, range) was significantly greater in group 4 than in groups 1 (*p* < 0.001), 2 (*p* < 0.001), and 3 (*p* = 0.005) (Figure 4a). The total abnormal MG ratio was significantly higher in group 4 than in groups 1 (*p* < 0.01) and 2 (*p* = 0.01) (Figure 4b). The thickened MG ratio was significantly greater in group 4 than in groups 1, 2, and 3 (*p* < 0.001) (Figure 4c). The shortened and distorted MG ratios were not significantly different among the four LMT groups (*p* > 0.05).

**TABLE 4:** Median (range) of meibomian gland loss rate and abnormal meibomian gland ratio of eyelid margin thickness divided into 4 grades.

	LMT 1 (≤ 1.00 mm)	LMT 2 (1.01–1.10 mm)	LMT 3 (1.11–1.25 mm)	LMT 4 (> 1.25 mm)
Abnormal MG (%)	79.2	91.7	91.3	95.7
MGLR (%)	5.50 (0–74)	3.50 (0–77)	14 (0–59)	37 (0–100)
Abnormal MG ratio	0.18 (0–1.5)	0.21 (0–0.85)	0.2 (0–0.75)	0.41 (0.2–1.5)
Thickened MG ratio	0 (0–0.14)	0 (0–0.28)	0.08 (0–0.25)	0.17 (0–0.50)
Shortened MG ratio	0.04 (0–0.4)	0.05 (0–0.57)	0.14 (0–0.50)	0.16 (0–1.0)
Distorted MG ratio	0.07 (0–1.5)	0 (0–0.3)	0.07 (0–0.3)	0.08 (0–0.40)

Abbreviations: LMT, eyelid margin thickness; MG, meibomian gland; MGLR, meibomian gland loss rate.

To estimate the threshold of eyelid thickness to indicate moderate MG loss (≥ meiboscore 2), the analysis using ROC curve revealed that MGD was indicative when the eyelid margin thickness was 1.20 mm or more (sensitivity = 0.645, specificity = 0.768, positive predictive value = 0.553, negative predictive value = 0.839, Youden's index = 0.413, AUC = 0.783).

## 4 Discussion

In this study, the definition of the LMT was adopted from humans, with the posterior lid margin revised as the gray line. The gray line is a more defined anatomical structure that is easier to visualize and situated in close proximity to the mucocutaneous junction. In the presence of MGD, the gland orifices may shift anteriorly or posteriorly; however, a shift of one or two orifices did not affect the setting of the lid margin, as it was defined as an extension of the overall trend.

In humans, some studies have used specialized equipment to measure LMT, such as anterior segment optical coherence tomography (AS‐OCT) and keratographs, or directly measured it with iron calipers, such as Vernier micrometer [22, 28, 29]. In this study, the LMT was measured on the slit lamp image. The advantages of this approach were that it was noninvasive, time‐efficient, and straightforward, as it could be conducted concurrently with slit lamp biomicroscopy without the need for special equipment, and it does not cause unnecessary discomfort or lengthy physical restraint of dogs. However, there was a potential for error depending on the degree of eyelid everting; over‐everting the eyelid might result in stretching and an increase in length, whereas under‐everting the eyelid might lead to a poorly visualized or distorted gray line in the image, resulting in underestimation [22]. Therefore, it was essential to ensure an adequate amount of eyelid eversion without distortion. To minimize the potential for error, the same researcher everted the eyelid at an appropriate level during slit lamp biomicroscopy.

In this study, the LMT was thicker in the age group over 12 years than in the age group under 8 years. In humans, studies have suggested that LMT increases with age, but it is unclear if this is solely due to biological changes [22, 30]. Both dogs and humans are known to have increased incidence of MGD with age [5, 19, 31]. In this study, the increased LMT observed in age group 4 is likely attributable to the higher prevalence of MGD cases in this group (92%) compared to the overall population (73%). Furthermore, also within age group 4, MGD dogs had a thicker mean LMT of 1.26 mm, compared to 1.08 mm in normal dogs. Due to the small number of normal dogs in group 4, further studies with larger sample sizes are needed to clarify the relationships between age, LMT, and MGD [32]. One limitation is that the different life stages between large and small breeds, even at the same age, were not considered in the analysis due to the small number of large breed dogs included in the study.

The LMT was not significantly different between the sexes, as in human studies, but intact females had a thinner LMT than spayed females. Some human studies have shown that sex hormones could affect MG lipid production [13, 21, 33, 34]; however, it was difficult to evaluate whether sex or spaying/neutering had an effect on LMT, with a limited number of dogs included in the study.

Meibography showed that the MGLR and abnormal MG ratios were greater in the MGD group than in the normal group, except for the distorted MG ratio. MG loss is an important indicator for the diagnosis of MGD; however, human studies have also shown that abnormal MG morphology, such as thickening, shortening and distortion, was associated with MGD [17, 26, 31].

In this study, evaluation of meibography according to LMT revealed that the MGLR, total abnormal MG ratio, and thickened MG ratio were significantly higher in the LMT group 4 (> 1.26 mm). Hyperkeratinization and obstruction of the MG orifice leads to MG dilation and thickening during the initial phase [7]. As this progresses to cystic degeneration and gland atrophy, remaining MGs thicken as a compensatory response to decreased secretion and increased demand for meibum. However, this compensatory mechanism eventually fails, resulting in gradual gland loss [34]. These histopathological changes in MG appear to be associated with LMT and the progression of MGD. Human studies have demonstrated that lid margin thickening correlates with a higher MGLR and an increased ratio of thickened MGs [9, 18]. This is consistent with the findings of this study that MG thickening and gland loss are exacerbated as LMT increases.

There were no significant differences in shortened or distorted MG ratios between the LMT groups. Shortened MG may or may not be observed depending on the progression of MGD, as it is considered a histologically atrophic change that could eventually lead to dropout in a human study [35].

Feng et al. suggested that distortion might be a transitional state between MG thickening and dropout, with a distorted MG negatively correlated with lid margin thickening [18]. However, MG distortion was only evaluated in patients with mild MG loss, and unlike shortening, distortion of the MG was not histologically different from normal MG in humans [34]. Thus, the relationship between MG distortion and LMT or MGD remains ambiguous. The mechanism of MG distortion is unclear, but previous human studies have suggested that chronic inflammation or chronic mechanical forces such as frequent eye rubbing due to conditions like allergic conjunctivitis might contribute [35, 36].

This study had several limitations. This study did not directly compare the MG morphology and LMT between the early and late stages of MGD. Changes in MG morphology have been studied in humans to diagnose MGD at an early stage. However, more studies are required in the veterinary field, considering the variability of results in humans and the complex pathogenesis of MGD.

This study was also limited by the inclusion of a relatively small number of dogs. The impact of systemic diseases or ophthalmic medications was not considered, and LMT and meibography were only evaluated in the upper eyelid, given that the upper eyelid was considerably more accessible for examination than the lower eyelid.

Some studies in both the human and veterinary fields have demonstrated that MG loss in the lower eyelid was greater than that in the upper eyelid [31, 37, 38], although other studies have shown that it was correlated between the upper and lower eyelids [32]. Nevertheless, further research is required to determine which eyelid should be considered a representative sample of MGD. The diagnosis of MGD was based only on the observation of eyelid abnormalities; the relationship between MGD and other ocular surface abnormalities, such as interferometry and NIBUT, was not investigated. The objective of this study was to analyze the correlation between LMT and meibography because LMT was an easily recognizable clinical feature.

In conclusion, LMT could be readily measured on slit lamp biomicroscopy images and correlated with abnormal MG morphology, especially thickening. LMT ≥ 1.20 mm was indicative of moderate MG loss. Therefore, the evaluation of LMT could be a simple screening tool to predict MG loss and could indicate the necessity for further examination, such as meibography, to confirm the diagnosis of MGD.

## Author Contributions

**Giyeon Kim:** conceptualization, formal analysis, investigation, methodology, project administration, software, visualization, writing – original draft. **Seonmi Kang:** investigation, supervision, writing – review and editing. **Junehee Seo:** data curation, visualization. **Kangmoon Seo:** funding acquisition, supervision, validation, writing – review and editing.

## Ethics Statement

This study was approved by the respective animal hospital and the Institutional Animal Care and Use Committee of Seoul National University (SNU‐240722‐5). In addition, the owners of the animals in this study consented to the clinical examination, treatment, and the use of related information in the publication.

## Conflicts of Interest

The authors declare no conflicts of interest.
